# Increase in the solubility of uvsY using a site saturation mutagenesis library for application in a lyophilized reagent for recombinase polymerase amplification

**DOI:** 10.1007/s11033-024-09367-y

**Published:** 2024-02-27

**Authors:** Kenta Morimoto, Kevin Maafu Juma, Masaya Yamagata, Teisuke Takita, Kenji Kojima, Koichiro Suzuki, Itaru Yanagihara, Shinsuke Fujiwara, Kiyoshi Yasukawa

**Affiliations:** 1 Division of Food Science and Biotechnology, Graduate School of Agriculture, Kyoto University, Kyoto, 606-8502 Japan; 2 Faculty of Pharmaceutical Sciences, Himeji Dokkyo University, Himeji, Hyogo 670-8524 Japan; 3 The Research Foundation for Microbial Diseases of Osaka University, Suita, Osaka 565-0871 Japan; 4 Department of Developmental Medicine, Research Institute, Osaka Women’s and Children’s Hospital, Izumi-Shi, Osaka, 594-1101 Japan; 5 Department of Biosciences, School of Biological and Environmental Sciences, Kwansei-Gakuin University, Sanda, Hyogo 669-1330 Japan

**Keywords:** Isothermal DNA amplification, Recombinase polymerase amplification (RPA), Site saturation mutagenesis library, uvsY

## Abstract

**Background:**

Recombinase uvsY from bacteriophage T4, along with uvsX, is a key enzyme for recombinase polymerase amplification (RPA), which is used to amplify a target DNA sequence at a constant temperature. uvsY, though essential, poses solubility challenges, complicating the lyophilization of RPA reagents. This study aimed to enhance uvsY solubility.

**Methods:**

Our hypothesis centered on the C-terminal region of uvsY influencing solubility. To test this, we generated a site-saturation mutagenesis library for amino acid residues Lys91–Glu134 of the N-terminal (His)_6_-tagged uvsY.

**Results:**

Screening 480 clones identified A116H as the variant with superior solubility. Lyophilized RPA reagents featuring the uvsY variant A116H demonstrated enhanced performance compared to those with wild-type uvsY.

**Conclusions:**

The uvsY variant A116H emerges as an appealing choice for RPA applications, offering improved solubility and heightened lyophilization feasibility.

**Supplementary Information:**

The online version contains supplementary material available at 10.1007/s11033-024-09367-y.

## Introduction

Recombinase polymerase amplification (RPA) amplifies a target DNA sequence at a constant temperature around 37–42 °C using recombinase (Rec), single-stranded DNA-binding protein (SSB), strand-displacing DNA polymerase (Pol), and ATP regenerating enzyme [1–3]. As shown in Fig. S1, the complex of Rec and primer binds to the target sequences in the DNA template. Then, SSB binds to the unwound strand and Pol extends the primer. After repeating this process, the target DNA fragment is synthesized. Since RPA was first reported in 2006 [1], uvsX and uvsY from T4 phage, gp32 from T4 phage, Pol from *Bacillus stearothermophilus* (*Bst-*Pol), and creatine kinase (CK) from rabbit have been consistently used as Rec, SSB, Pol, and ATP regenerating enzyme, respectively. Unlike PCR, RPA does not require a thermal cycler. Therefore, RPA is potentially suitable for the onsite detection of various pathogens. However, current commercial RPA kits require a storage in a deep freezer, which is inconvenient for onsite use of RPA. Lyophilized RPA reagent that can be stored at room temperature is highly desired.

**Fig. 1:**
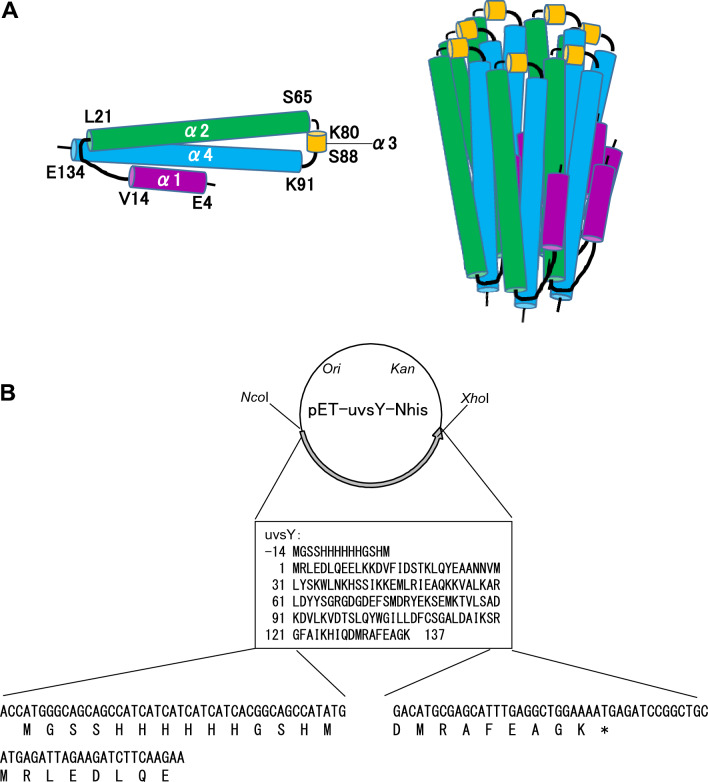
Structure of uvsY. (**A**) Tertiary structure of uvsY. (**B**) pET-uvsY-Nhis. The asterisk indicates the termination codon. Amino acid sequence of uvsY is shown

We first prepared recombinant uvsX, uvsY, and gp32 using the *Escherichia coli* expression system [4] and used them to examine the effects of additives on the RPA reaction efficiency [4] and to optimize the RPA reaction conditions using the statistical method [5]. Preparation of uvsX, uvsY, and gp32 revealed that the solubility of uvsY is lower than those of uvsX and gp32. When the uvsX, uvsY, and gp32 having a (His)_6_ tag and a thrombin recognition site at each of N and C terminus were treated with thrombin to cleave (His)_6_, uvsY became insoluble while uvsX and gp32 remained soluble [4]. We assessed the impact of (His)_6_-tag placement on RPA performance, observing higher reaction efficiency with N-terminal (His)_6_-tagged uvsY (uvsY-Nhis) compared to C-terminal (His)_6_-tagged (uvsY-Chis) or N- and C-terminal (His)_6_-tagged (uvsY-NChis) [6]. However, uvsY-Nhis exhibited lower solubility than that of uvsX and gp32. It is known that the original uvsY (137 amino acid residues) functions as a heptamer (Fig. 1A) [7–9].

Site saturation mutagenesis involves randomizing a set of codons to generate a variant library, with every possible amino acid at each randomized position in the target region [10–12]. Unlike error-prone PCR, this method avoids generating mutations unrelated to amino acid substitutions. We have used this technique to increase the thermostabilities of reverse transcriptase from Moloney murine leukemia virus [13] and GH10 xylanase, XynR, from *Bacillus* sp. strain TAR-1 [14]. In this study, we utilized site saturation mutagenesis to increase the solubility of uvsY.

## Materials and methods

### Materials

uvsY-Nhis was expressed in *E. coli* as described previously [6]. The uvsY concentration was determined using the molar absorption coefficient at 280 nm (ε_280_) of 19,940 M^−1^ cm^−1^. uvsX and gp32 were expressed in *E. coli*, as N- and C-terminal (His)_6_-tagged proteins with a thrombin recognition site, and their (His)_6_ s were removed by thrombin treatment as described previously [4]. The concentrations of uvsX and gp32 were determined using ε_280_ of 33,015 and 41,160 M^−1^ cm^−1^, respectively. DNA polymerase (H1-Pol) from a thermophilic bacterium *Aeribacillus pallidus* (H1) was expressed in *E. coli* as N-terminal (His)_6_-tagged protein as described previously [15]. The H1-Pol concentration was determined by the method of Bradford using Protein Assay CBB Solution (Nacalai Tesque, Kyoto, Japan) with bovine serum albumin (Nacalai Tesque) as standard. Rabbit CK was purchased from Roche (Mannheim, Germany). Human pyruvate kinase (PK) was expressed in *E. coli* as described previously [16]. The PK concentration was determined using ε_280_ of 30,410 M^−1^ cm^−1^.

### Construction of site saturation mutagenesis library

The site saturation mutagenesis libraries were constructed using Quikchange HT Protein Engineering System (Agilent Technologies) as follows: The oligonucleotide set targeting Lys91–Glu134 was designed and synthesized by Agilent Technology with a microarray technology. The oligonucleotide set was amplified by PCR. Precisely, the reaction solution (50 µL) was prepared by mixing 14 µL of water, 1 µL of QuikChange HT Mutagenesis Library solution (Agilent Technologies), 25 µL of 2 × PfuUltra II HS Master Mix AD (Agilent Technologies), 5 µL of 5 µM uvsY3 primer (5´-CAGCCGGATCTCATTTTCCAGC-3´), and 5 µL of 5 µM uvsY5 primer (5´-TCAGAAATGAAGACAGTTCTATCAGCGG-3´). The thermal cycling condition was 30 s at 95 °C, 30 cycles of 20 s at 95 °C, 10 s at 60 °C, and 30 s at 72 °C, and 2 min at 72 °C. The amplified oligonucleotide set was purified using StrataPrep PCR Purification Kit (Agilent Technologies). The plasmid library was constructed by PCR using QuikChange Lightning Site-Directed Mutagenesis Kit (Agilent Technologies) with pET-uvsY-Nhis (1 ng/µL) (Fig. 1B) [6] as a template and the amplified oligonucleotide set as primers. The thermal cycling condition was 2 min at 95 °C, 18 cycles of 20 s at 95 °C, 10 s at 60 °C, and 5 min at 68 °C, and 5 min at 68 °C. Amplified products were transfected into *E. coli* strain BL21(DE3) [*F*^−^, *ompT*, *hsdS*_*B*_ (*r*_*B*_^−^ *m*_*B*_^−^) *gal dcm* (DE3)] (Takara Bio, Kusatsu, Japan).

### Screening of uvsY variants with an increased solubility using 96-well plates

(i) Expression of site saturation mutagenesis library and preparation and characterization of soluble and insoluble fractions: A colony of the transformed *E. coli* cells was inoculated in LB broth (1 mL) containing 100 µg/mL kanamycin in a 96-well deep plate, and the culture was incubated at 30 °C for 20 h with shaking. The optical density at 600 nm (*OD*_600_) of the culture was adjusted to be 0.5 by diluting with LB broth containing 100 µg/mL kanamycin. Then, isopropyl-β-D-1-thiogalactopyranoside (IPTG) was added for the final concentration to be 1 mM. The culture was continued at 20 °C for 24 h with shaking. After centrifugation (3000×*g*, 10 min, 4 °C), the cell pellets were collected, suspended in 100 µL of 20 mM Tris–HCl buffer (pH 8.0), 0.2 M NaCl and disrupted by sonication. After centrifugation (20,000×*g*, 10 min, 4 °C), the supernatants (soluble fraction) and pellets (insoluble fraction) were collected.

(ii) SDS-PAGE analysis of the soluble and insoluble fractions: The soluble and insoluble fractions (8 µL) were mixed with the SDS-PAGE sample buffer (2 µL of 0.25 M Tris–HCl buffer (pH 6.8), 50% v/v glycerol, 10% w/v SDS, 5% v/v 2-mercaptoethanol, 0.05% w/v bromophenol blue) and were heated at 100 °C for 10 min. The solution (10 µL) was applied to 15% polyacrylamide gel with a constant current of 40 mA for 40 min. After electrophoresis, gels were stained with 0.25% Coomassie Brilliant Blue R-250, 50% methanol, and 7% acetic acid.

### Sequence analysis

To identify the mutation, the entire nucleotide sequence of the uvsY gene of the plasmids were determined by the dideoxynucleotide chain termination procedure using the T7 promoter primer (5´-TAATACGACTCACTATAGGG-3´).

### Expression and purification of uvsY

The overnight culture of the transformants (60 mL) was added to 600 mL of LB broth containing 50 μg/mL kanamycin and incubated with shaking at 37 °C. When *OD*_600_ reached 0.6–0.8, the culture (600 mL) was added to 2000 mL of LB broth containing 50 μg/mL kanamycin, and 0.5 M IPTG was added for the final concentration to be 0.5 mM. The culture was continued at 30 °C for 4 h. The cells were harvested by the centrifugation of the culture at 3000×*g* for 10 min and suspended with 50 mL of 50 mM phosphate buffer (pH 7.2), 1 M NaCl, 2 mM phenylmethylsulfonyl fluoride (PMSF), and disrupted by sonication. After centrifugation (20,000×*g*, 4 °C, 20 min), the supernatant was collected as the soluble fraction of the cells.

Solid (NH_4_)_2_SO_4_ was added to the soluble fraction of the cells to a final concentration of 40% saturation. After centrifugation (10,000×*g*, 10 min, 4 °C), the supernatant was collected and adjusted to a final concentration of 60% saturation. Following the centrifugation, the pellet was dissolved in 50 mL of 50 mM potassium phosphate buffer (pH 7.2), 0.5 M NaCl (buffer A). The solution was applied to the column packed with a Ni^2+^-sepharose (Profinity IMAC resin 5 mL, BioRad, Hercules, CA) equilibrated with buffer A. After washing with 100 mL of buffer A containing 20 mM imidazole, the bound enzyme was eluted with 30 mL of buffer A containing 600 mM imidazole. Each fraction was assessed for the presence of enzyme by 12.5% SDS-PAGE. The active fractions were collected, concentrated to 1 mL in 10 mM Tris–HCl buffer (pH 8.0), 0.5 M NaCl, 600 mM imidazole by Amicon Ultra-15 MWCO 10 k (Merck Millipore, Burlington, MA), and stored in 7.5 mM Tris–HCl buffer (pH 8.0), 0.375 M NaCl, 450 mM imidazole, 20% v/v glycerol at − 30 °C.

### Preparation of standard DNA

The 233-bp DNA fragment of the urease subunit β (UreB) gene from *Ureaplasma parvum* serovar 3, corresponding to DNA sequence 519–751 deposited in GenBank (AF085732.1), was amplified by PCR using primers UreB28F3 and UreB260R3 (Fig. S2) and *Taq* polymerase (Toyobo, Osaka, Japan) under 35 cycles of 30 s at 95 °C, 30 s at 55 °C, and 30 s at 72 °C, and purified using MagExtractor (Toyobo). The concentration of purified DNA was determined spectrophotometrically at *A*_260_ and stored at −20 °C for subsequent use.

### RPA reaction

Target region for primers annealing for the RPA detection system for ureaplasma is shown in Fig. S2. The reaction volume was 30 µL, and the reaction condition was 400 ng/µL uvsX, 40 ng/μL uvsY, 600 ng/µL gp32, 40 ng/μL H1-Pol, 120 ng/µL CK, 2 mM DTT, 6% w/v PEG35000, 3.5 mM ATP, 650 μM dNTPs, 50 mM Tris–HCl buffer (pH 8.6), 40 mM CH_3_COOK, 20 mM phosphocreatine, 14 mM Mg(OCOCH_3_)_2_, 1 µM UreB28F3 primer, 1 µM UreB260R3 primer at 41 °C. The reaction was performed in a 0.2 ml PCR tube in PCR Thermal Cycler Dice (Takara Bio). The amplified products were separated on 2.0% (w/v) agarose gels and stained with ethidium bromide (1 μg/mL).

### Lyophilization of RPA reagents

The following solutions were mixed: 66 µL of 18.28 mg/mL uvsX in 20% w/v glycerol, 10 mM Tris–HCl (pH 8.0); 96 µL of 3.79 mg/mL wild-type uvsY or 30 µL of 12 mg/mL uvsY variant A116H in 20% w/v glycerol, 10 mM Tris–HCl (pH 8.0); 110 µL of 16.65 mg/mL gp32, in 20% w/v glycerol, 10 mM Tris–HCl (pH 8.0); 14 µL of 1.7 mg/mL H1-Pol in 50% glycerol, 50 mM KCl, 1 mM DTT, 0.1 mM EDTA, 0.1% Triton® X-100, 10 mM Tris–HCl buffer (pH 7.1); and 14 µL of 4.3 mg/mL human PK in 20% w/v glycerol, 10 mM Tris–HCl (pH 8.0). The mixed solution was applied to PD-10 gel filtration column (GE Healthcare, Buckinghamshire, UK) equilibrated with 25 mL of 10 mM CH_3_COONH_4_ buffer (pH 6.0). After sample application, the column was washed with 2.0 mL of 10 mM CH_3_COONH_4_ buffer (pH 6.0). The enzymes were eluted with 2.0 mL with the same buffer as above. To the fraction, the following solutions were added: 75 µL of 2 M Tris–HCl (pH 8.6), 40 µL of 3 M CH_3_COOK, 900 µL of 20% w/v PEG35000, 50 µL of 120 mM dithiothreitol, 78 µL of 25 mM dNTPs in water, 100 µL of 30 µM DNA primer UreB28F3, 100 µL of 30 µM DNA primer UreB260R3, 30 µL of 0.5 M phosphoenolpyruvate, 70 µL of 150 mM ATP, 467 µL of 30% trehalose. The mixed solution was dispensed by 30 µL in a 0.2 mL PCR tube, frozen at −80 °C for 1 h, and then dried using EYELA-FD1000 (Tokyo Rikakikai, Tokyo, Japan) freeze dryer at 50 Pa, −50 °C, for 16 h.

### RPA reaction using the lyophilized reagent

The lyophilized reagent in the PCR tube was dissolved by adding water (27.9 μL). The reaction was started by adding 0.6 μL of the target DNA solution and 1.5 μL of 280 mM Mg(OCOCH_3_)_2_ and continued at 41 °C for 30 min. The amplified products were separated on 2.0% (w/v) agarose gels and stained with ethidium bromide (1 μg/mL).

## Results and discussion

### Construction of the site saturation mutagenesis library

**Fig. 2:**
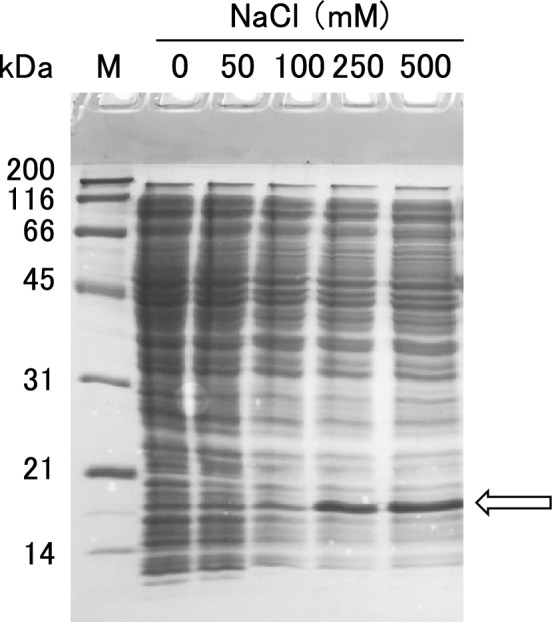
Effects of the NaCl concentration on the solubility of wild-type uvsY. Soluble fraction at 0–500 mM NaCl of the extract of the BL21(DE3) cells transformed with the wild-type uvsY expression plasmid and cultured at 20 °C for 24 h after IPTG induction was applied to 12.5% polyacrylamide gel. CBB-stained gel is shown

We initially investigated the solubility of uvsY. UvsY was expressed in *E. coli* BL21(DE3) transformed with the pET-uvsY-Nhis plasmid (Fig. 1B). The NaCl concentration of the cell suspension was adjusted to 0, 50, 100, 250, or 500 mM, and both soluble and insoluble fractions were prepared. Figure 2 depicts the SDS-PAGE of the soluble fractions. This indicates that a NaCl concentration of 250 mM or more is necessary to prevent the insolubilization of uvsY. Considering the performance of RPA reagents, an increase in the solubility of uvsY is desirable.

**Fig. 3:**
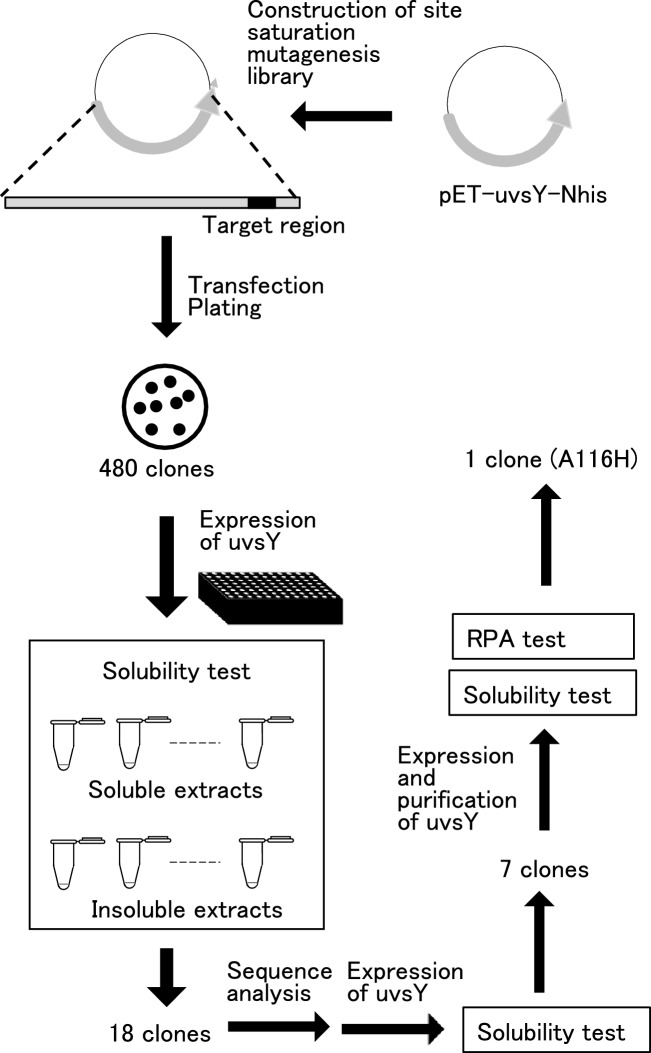
Illustration of workflow. The workflow to construct a site saturation mutagenesis library and to screen uvsY variant with high solubility and results are shown

Solubility is a crucial characteristic of proteins, yet what determines protein solubility is not well understood. Several reports have suggested that single amino acid mutations can significantly alter protein solubility [17–19]. Crystallographic analysis of uvsY revealed that uvsY exists as a heptamer, with one uvsY molecule consisting of four α-helices (α1–α4: α1, Glu4–Va14; α2, Leu21–Ser65; α3, Lys80–Ser88; and α4, Lys91–Glu134) (Fig. 1A) [9–11]. When viewed from the top of the heptamer, α1, α2, and α3 are located externally, whereas α4 is positioned internally. In one heptamer, seven N-terminal residues are distantly spaced, and seven C-terminal residues are closely positioned. We hypothesized that the C-terminal region of uvsY is responsible for its solubility. We selected Lys91–Glu134 (44 amino acids) to cover the C-terminal region of uvsY and constructed a site saturation mutagenesis library corresponding to this region (Fig. 3).

### Expression of the site saturation mutagenesis library and screening of uvsY variants with an increased solubility

The library theoretically comprised 880 clones (44 amino acids × 20; 880 clones). Due to the impracticality of evaluating over a thousand clones individually, we assessed 480 clones. As shown in Fig. 3, in a 96-well plate, both wild-type uvsY (WT) and the 480 clones were expressed in *E. coli*. The soluble and insoluble fractions of *E. coli* cells were prepared from both WT and the 480 clones. SDS-PAGE results of the soluble and insoluble fractions for each clone are depicted in Fig. S3. We identified 18 clones with higher solubility than WT. Notably, among these 18 clones, 3H11 exhibited a higher molecular mass (25 kDa) compared to the other 17 clones (18 kDa).

### Sequence analysis

**Fig. 4:**
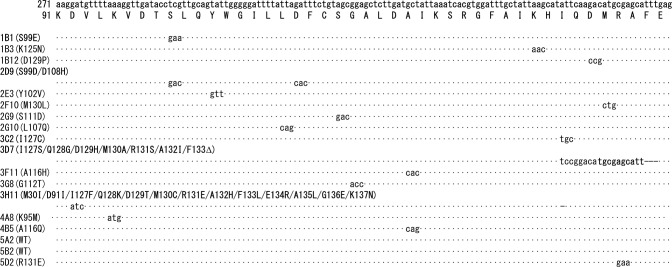
Sequence analysis of uvsY variants. Substitution of nucleic acid residue is indicated by characters. Deletion of nucleic acid residue is indicated by “-”

We analyzed the full sequence of the 18 clones. Figure 4 shows the results of sequence corresponding to Lys91–Glu134. In site saturation mutagenesis library, it is expected that in each clone, one amino acid residue in a target region is substituted into other 19 amino acid residues. Thirteen clones (72%) had expected mutations corresponding to one amino acid substitution (1B1, 1B3, 1B12, 2E3, 2F10, 2G9, 2G10, 3C2, 3F11, 3G8, 4A8, 4B5, and 5D2). Three clones (17%) had unexpected mutations [two mutations for 1 clone (2D9); six amino acid substitutions and one amino acid deletion for 1 clone (3D7), and one amino acid substitution and one nucleotide deletion-derived frame shift for 1 clone (3H11)]. This may have been caused by errors in oligonucleotides synthesis in the microarray. Two clones (11%) had no mutation (5A2 and 5B2). In four of the variants, hydrophobic amino acid residues, Leu, Ile, Ala, and Ala were replaced with the hydrophilic amino acid residues, Gln, His, Thr, and Gln, respectively.

### Solubility test

**Fig. 5:**
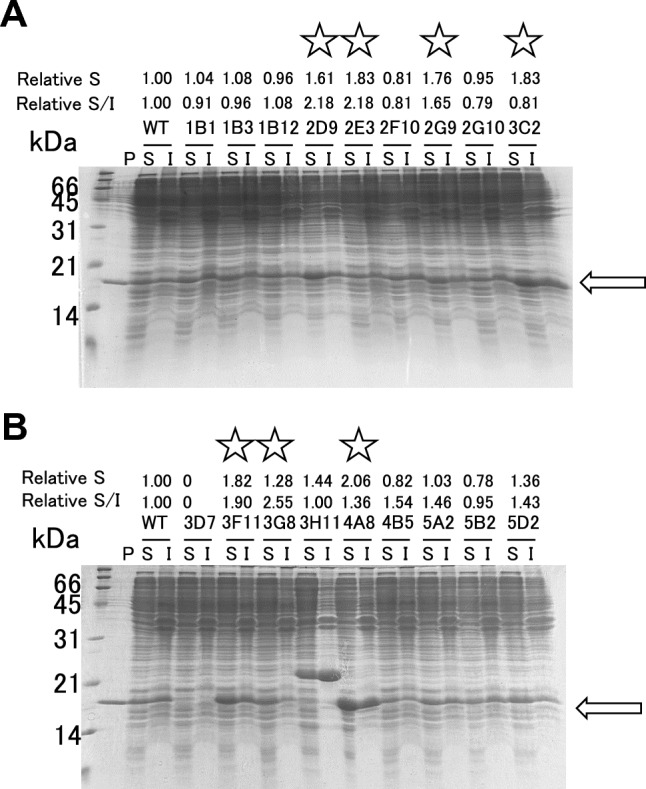
Solubility of uvsY variants. CBB-stained 15% polyacrylamide gels are shown. P indicates the purified uvsY. S and I indicate soluble and insoluble fractions of the extract of the BL21(DE3) cells transformed with uvsY variants expression plasmid and cultured at 20 °C for 24 h after IPTG induction. S indicates the intensity of the band corresponding to soluble fraction by imageJ analysis. S/I indicates the ratio of the intensity of the band corresponding to soluble fraction to that corresponding to insoluble fraction by imageJ analysis. Relative S and S/I indicate the values compared to WT. The variants whose amounts in the soluble fraction were comparable to or more than that in the insoluble fraction are marked with a star

Figure 5 depicts SDS-PAGE results for the 18 clones, showing soluble and insoluble fractions. Using ImageJ [20], we quantified band intensities in both fractions, calculating ratios. Seven clones (Y102V, S99D/D108H, S111D, I127C, G112T, A116H, and K95M) were chosen for further analysis based on these results.

### Purification of uvsY variants and their solubility test

**Fig. 6:**
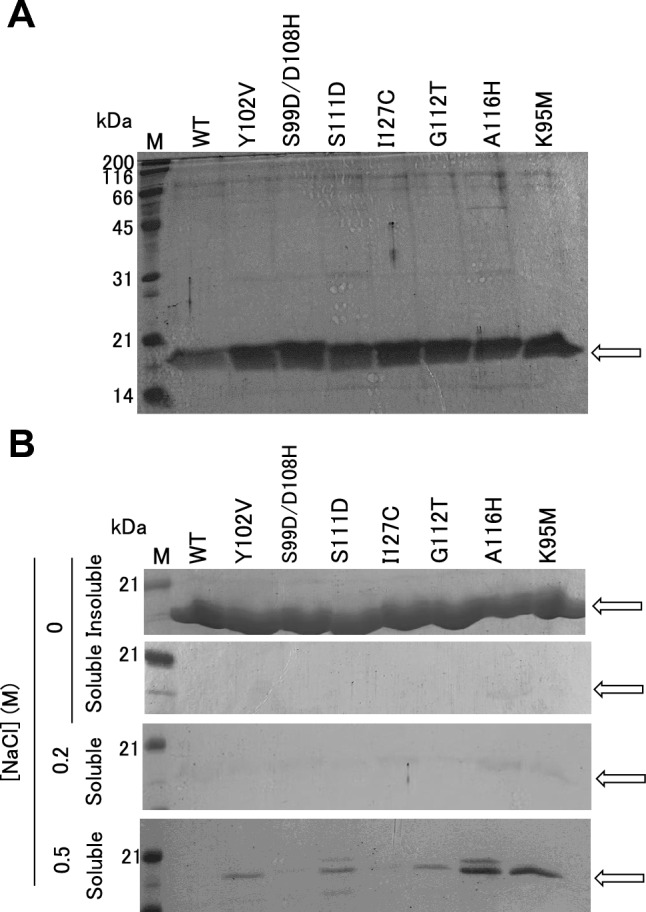
Purification and characterization of uvsY variants. CBB-stained 15% polyacrylamide gel is shown. (**A**) Purified preparations. (**B**) Effects of the NaCl concentration on the solubility of uvsY variants. Purified uvsYs were dialyzed against 0, 0.2, or 0.5 M NaCl at 4 °C followed by centrifugation (20,000×*g*, 4 °C, 10 min). The supernatant and precipitates were collected and subjected to SDS-PAGE

Purification of the WT and seven uvsY variants was carried out following a previously described procedure [6]. From a 2 L culture, we obtained 5–8 mg of both WT and variants. The results of the SDS-PAGE analysis of the purified preparations are illustrated in Fig. 6A, showing single bands with a molecular mass of 18 kDa for both the WT and the seven variants.

To assess solubility, we prepared soluble and insoluble fractions using various NaCl concentrations, as depicted in Fig. 6B. At 0.5 M NaCl, the band corresponding to uvsY was present in the soluble fractions of A116H and K95M but not in the WT or the other five clones. At 0 M and 0.2 M NaCl, clear bands did not appear in either the WT or any of the seven variants. These results suggest that the solubilities of A116H and K95M were higher than those of the WT or the other five clones.

### Effects of uvsY concentration on the RPA reaction efficiency

In RPA, uvsX binds to DNA primer in the presence of ATP to form the nucleoprotein with the aid of uvsY. When ATP is hydrolyzed, uvsX dissociates from DNA primer and is replaced by gp32. Therefore, the balance of the binding and dissociation between uvsX and DNA primer should be adequately controlled. If this binding affinity is too high, uvsX does not dissociate even after the elongation starts, preventing another nucleoprotein from binding to the target sequence and starting the elongation. On the other hand, if this binding affinity is too weak, the nucleoprotein cannot invade double-stranded DNA.

**Fig. 7:**
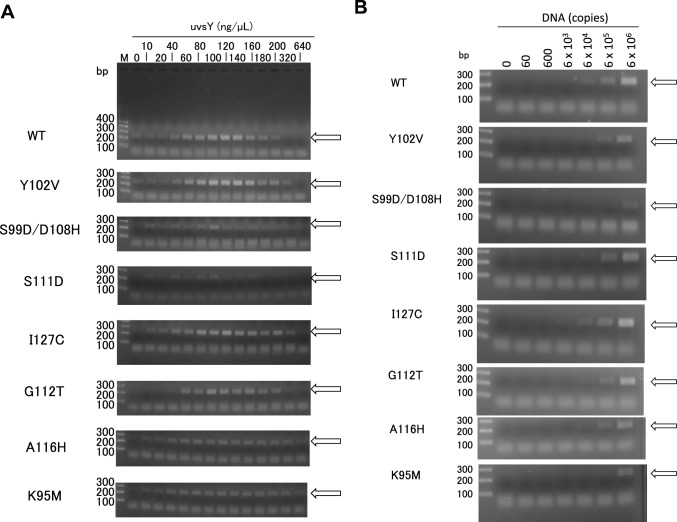
Characterization of uvsY variants in RPA. (**A**) Effects of the uvsY concentration on the reaction efficiency of RPA. The reactions were carried out with 0–640 ng/μL wild-type uvsY or its variants at 41 °C for 30 min. The initial copy of standard DNA was 6 × 10^5^. The arrow indicates the 233-bp target band. (**B**) Effects of initial copies on the RPA reaction. The reactions were carried out with the standard DNA of 0–6 × 10^6^ copies at 41 °C for 30 min. The uvsY concentrations were 80 ng/μL for S111D, 100 ng/μL for S99D/D108H and G112T, 120 ng/μL for WT, Y102V, I127C, and A116H, and 140 ng/μL for K95M. The arrow indicates the 233-bp target band

We evaluated the effects of the uvsY concentrations on the RPA reaction efficiency for their performances in RPA reaction. For this purpose, we used the detection system of *ureB* gene of ureaplasma, a bacterium that is commonly found in people’s urinary or genital tract [21–23]. The sequences of target gene and forward and revere primers are shown in Fig. S2. The size of amplified product was 233 bp. RPA was carried out with the initial copy number of 6 × 10^5^ copies of standard DNA at 41 °C for 30 min. Figure 7A shows the agarose gel analysis of the amplified RPA products. The RPA with WT exhibited the target DNA band clearly at the WT concentrations of 60–180 ng/µL. The RPAs of Y102V, I127C, G112T, A116H, or K95M exhibited the similar results as WT. The RPAs of S99D/D108H or S111D exhibited the target band, however it was less clear. Based on these results, we set 80 ng/μL for S111D, 100 ng/μL for S99D/D108H and G112T, 120 ng/μL for WT, Y102V, I127C, and A116H, and 140 ng/μL for K95M as the concentration of uvsY for subsequent analysis.

### Sensitivities of the RPA reactions with uvsY variants

We examined the sensitivities of RPA reaction with uvsY variants. RPA was performed with initial copy number of 0–6 × 10^6^ copies of standard DNA at 41 °C for 30 min. Figure 7B shows the agarose gel analysis of the amplified RPA products. The RPA with WT exhibited the target band at the initial copy numbers of 6 × 10^4^ copies or more. The RPAs of I127C, G112T, or A116H exhibited the similar results as WT. The RPAs of Y102V, S99D/D108H, S111D, or K95M exhibited the target band at the initial copy numbers of 6 × 10^5^ copies or more. These results indicated that the RPA reaction with I127C, G112T, or A116H was as sensitive as that with WT.

### Performance of lyophilized RPA reagents

Based on the results above described and shown in Figs. 6B and 7A, B we selected A116H, prepared the lyophilized RPA reagent with this variant, and compared its performance with the lyophilized RPA reagent with WT. As we previously described [6], preparation of lyophilized reagents used for RPA reagents consists of three steps: (1) Removal of glycerol present in enzyme preparation by gel filtration chromatography; (2) preparation of a mixture of all the reagents, except for Mg(OCOCH_3_)_2_ solution; and (3) lyophilization of the mixture using 4% w/v trehalose.

**Fig. 8:**
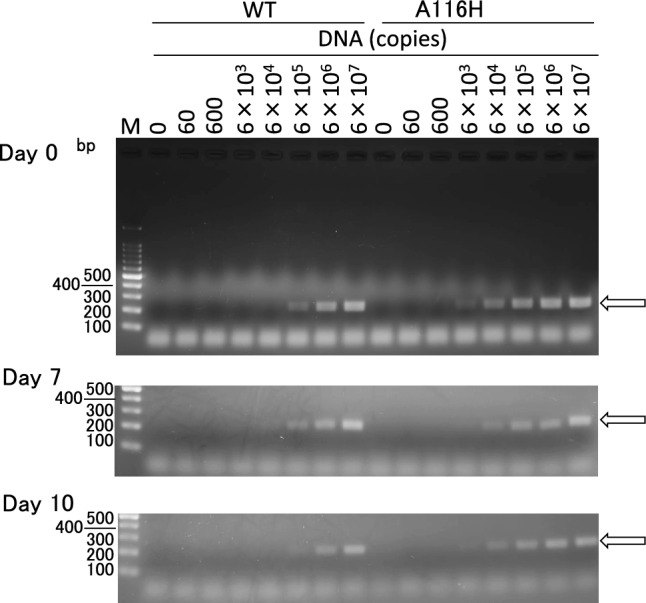
Effects of initial copies on the RPA reaction by lyophilized reagents. The lyophilized reagents containing the wild-type uvsY or its variant A116H were stored at 20 °C for 0–10 days. The reactions were carried out at 41 °C for 30 min. Initial copies of standard DNA were 0–6 × 10^7^. The arrow indicates the 233-bp target band

Lyophilized RPA reagents containing both WT and A116H were prepared, each equivalent to 100 tests. In each reagent, the concentrations of uvsX, gp32, and H1-Pol at the time of reaction were set as 400, 600, and 8 ng/μL, respectively, which were the same to those used for liquid reagents. After lyophilization, the reagents were dissolved in water, and their performance was evaluated. RPA was conducted with an initial copy number ranging from 0 to 6 × 10^7^ copies of standard DNA at 41 °C for 30 min (Fig. 8). The lowest initial copy number at which the amplified band was observed was 6000 copies for the RPA reagents containing A116H, whereas, for the WT, it was 6 × 10^5^ copies, indicating higher sensitivity in the former reagents. Subsequently, we assessed the storage stability of lyophilized RPA reagents stored at 20 °C. As illustrated in Fig. 8, on days 7 and 10, the lowest initial copy number at which the amplified band appeared in the RPA with A116H was lower than that with the WT. These results suggest that lyophilized RPA reagents containing A116H exhibit good storage stability. This implies that in the lyophilized RPA reagent containing WT, a certain portion of the WT did not dissolve in water, while in the reagent containing A116H, A116H performed better than WT.

### Limitation

The present study demonstrates that after screening 480 clones, A116H was selected as the variant with the highest solubility. Considering that the number of clones was limited, our results does not deny the presence of more soluble clones.

## Conclusions

A uvsY variant with increased solubility was obtained by screening a site saturation mutagenesis library. The lyophilized RPA reagent prepared with this uvsY variant was more sensitive than the reagent prepared with wild-type uvsY. These results suggest that this variant is attractive for use in RPA. Our results pave the way for use of RPA for point-of-care use in various fabrications.

## Supplementary Information

Below is the link to the electronic supplementary material.

Supplementary file1 (PDF 1810 KB)

## Data Availability

All data are available in case of need.

## Abbreviations

RPA: Recombinase polymerase amplification
Rec: Recombinase
SSB: Single-stranded DNA-binding protein
Pol: Strand-displacing DNA polymerase
PK: Pyruvate kinase
