# Supplementary material for: Increase in the solubility of uvsY using a site saturation mutagenesis library for application in a lyophilized reagent for recombinase polymerase amplification
Source: Mol Biol Rep. 2024 Feb 27;51(1):367. doi: 10.1007/s11033-024-09367-y (PMC10899321; doi:10.1007/s11033-024-09367-y)
Supplement: Supplementary file 1 — Supplementary file1 (PDF 1810 KB) [file 11033_2024_9367_MOESM1_ESM.pdf]

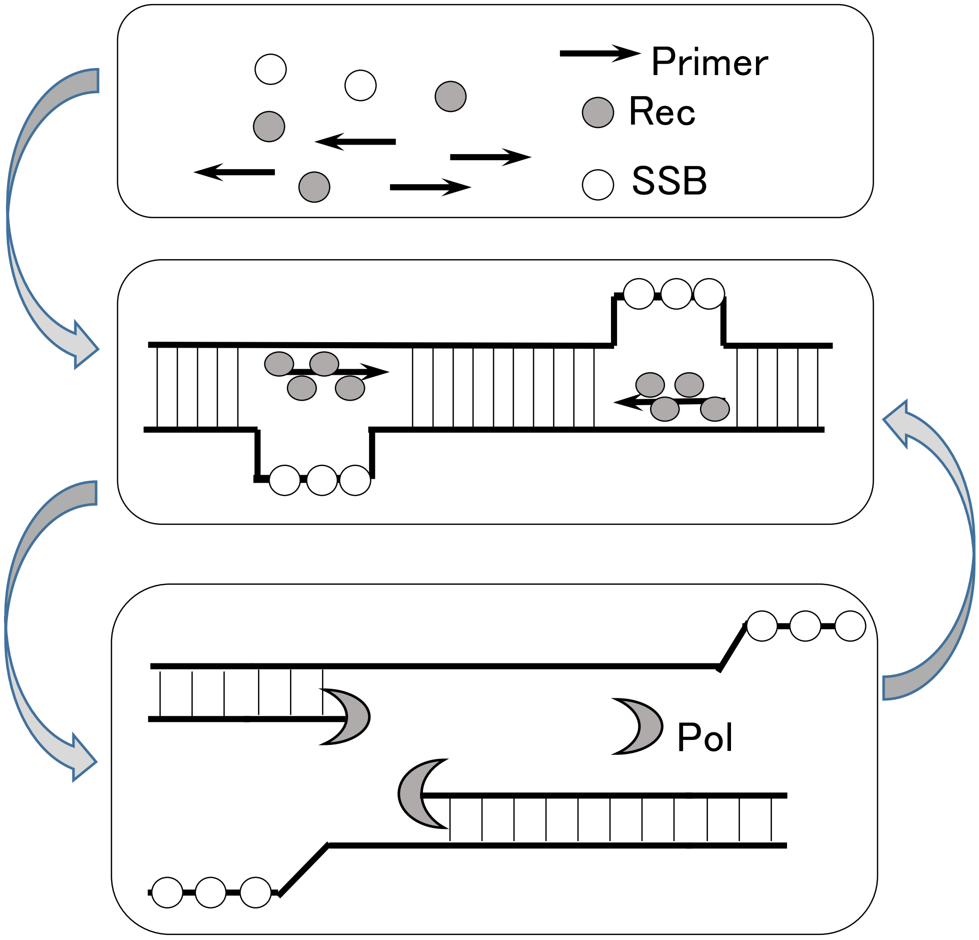

Fig. S1. Mechanism of RPA.
Rec, SSB, and Pol indicate recombinase, single-stranded DNA-binding protein, and strand-displacing DNA polymerase, respectively.

```
                                  UreB28F3
                           ------------------->
  1  atgtcaggatcatcaagtcaatttagtccaggtaaattagtaccaggggcaattaatttcgctagtggtgaaattgtgatgaatgaaggt
  1  M  S  G  S  S  S  Q  F  S  P  G  K  L  V  P  G  A  I  N  F  A  S  G  E  I  V  M  N  E  G

 91  agagaggcaaaagtaattagtattaaaaatactggggaccgtcctatacaagttggatcacattttcacttgtttgaagtgaatagtgca
 31  R  E  A  K  V  I  S  I  K  N  T  G  D  R  P  I  Q  V  G  S  H  F  H  L  F  E  V  N  S  A

181  ttagtattttttgatgaaaaaggaaatgaagataaagaacgcaaagttgcttatggacgacgtttcgatattccatcaggtactgctatt
 61  L  V  F  F  D  E  K  G  N  E  D  K  E  R  K  V  A  Y  G  R  R  F  D  I  P  S  G  T  A  I
                                                                 <--------------------
                                                                       UreB260R3

271  cgttttgaaccaggagataaaaaagaagtttcaattattgatttagccggaacacgcgaagtttgaggtgtaaatggcttagttaatgga
 91  R  F  E  P  G  D  K  K  E  V  S  I  I  D  L  A  G  T  R  E  V  -  G  V  N  G  L  V  N  G

361  aaacttaaaaaataa
121  K  L  K  K  -
```

Fig. S2. Nucleotide sequence of the target UreB DNA and the primers.

A

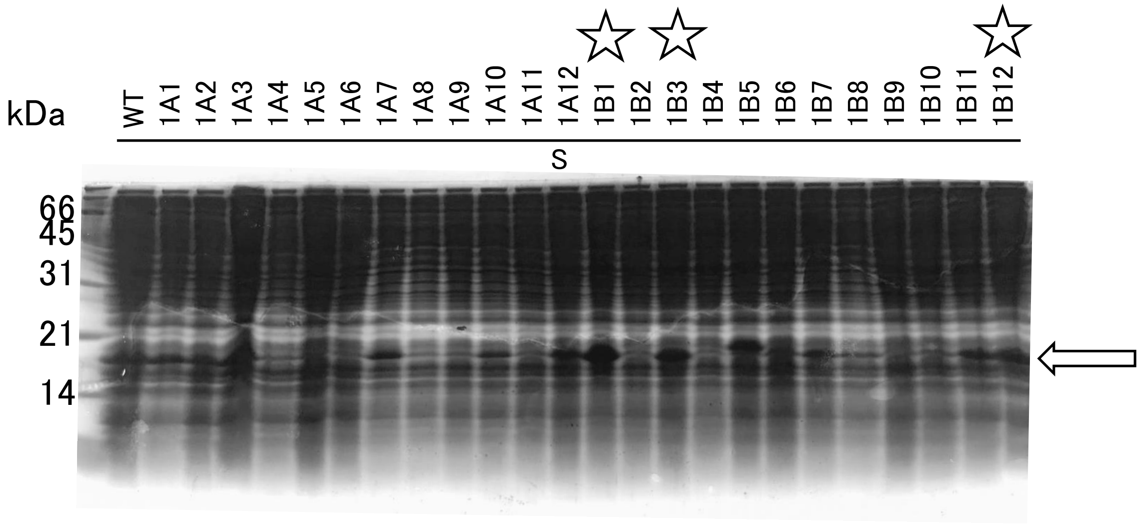
kDa
WT
1A1
1A2
1A3
1A4
1A5
1A6
1A7
1A8
1A9
1A10
1A11
1A12
1B1
1B2
1B3
1B4
1B5
1B6
1B7
1B8
1B9
1B10
1B11
1B12
S
66
45
31
21
14

B

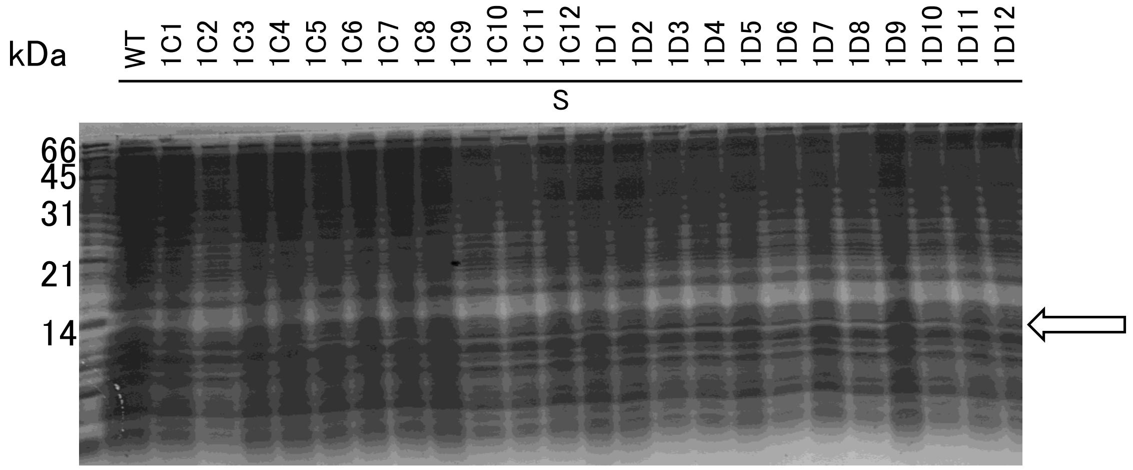
kDa
WT
1C1
1C2
1C3
1C4
1C5
1C6
1C7
1C8
1C9
1C10
1C11
1C12
1D1
1D2
1D3
1D4
1D5
1D6
1D7
1D8
1D9
1D10
1D11
1D12
S
66
45
31
21
14

Fig. S3

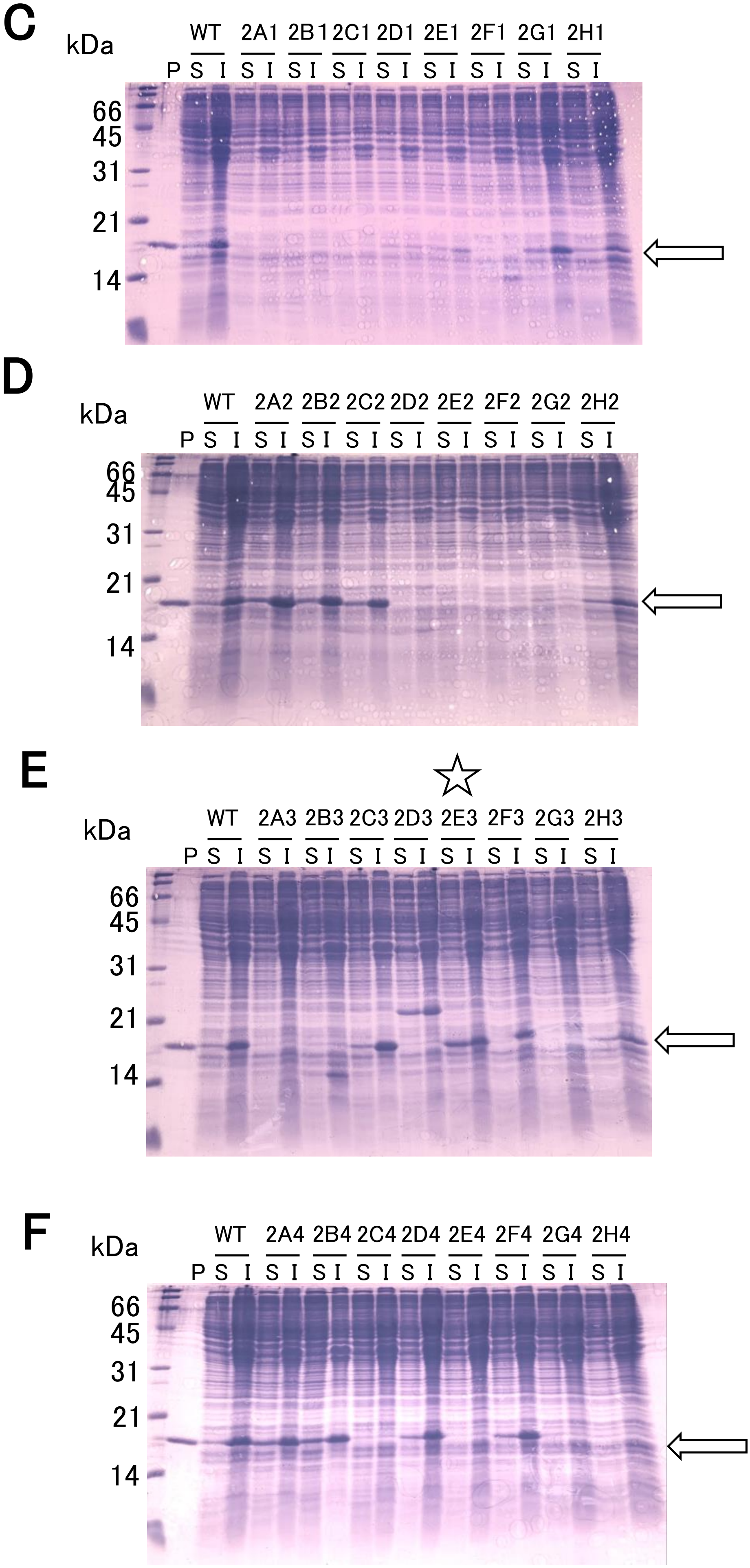

C
kDa
WT 2A1 2B1 2C1 2D1 2E1 2F1 2G1 2H1
P S I S I S I S I S I S I S I S I S I
66
45
31
21
14
D
kDa
WT 2A2 2B2 2C2 2D2 2E2 2F2 2G2 2H2
P S I S I S I S I S I S I S I S I S I
66
45
31
21
14
E
kDa
WT 2A3 2B3 2C3 2D3 2E3 2F3 2G3 2H3
P S I S I S I S I S I S I S I S I S I
66
45
31
21
14
F
kDa
WT 2A4 2B4 2C4 2D4 2E4 2F4 2G4 2H4
P S I S I S I S I S I S I S I S I S I
66
45
31
21
14

Fig. S3

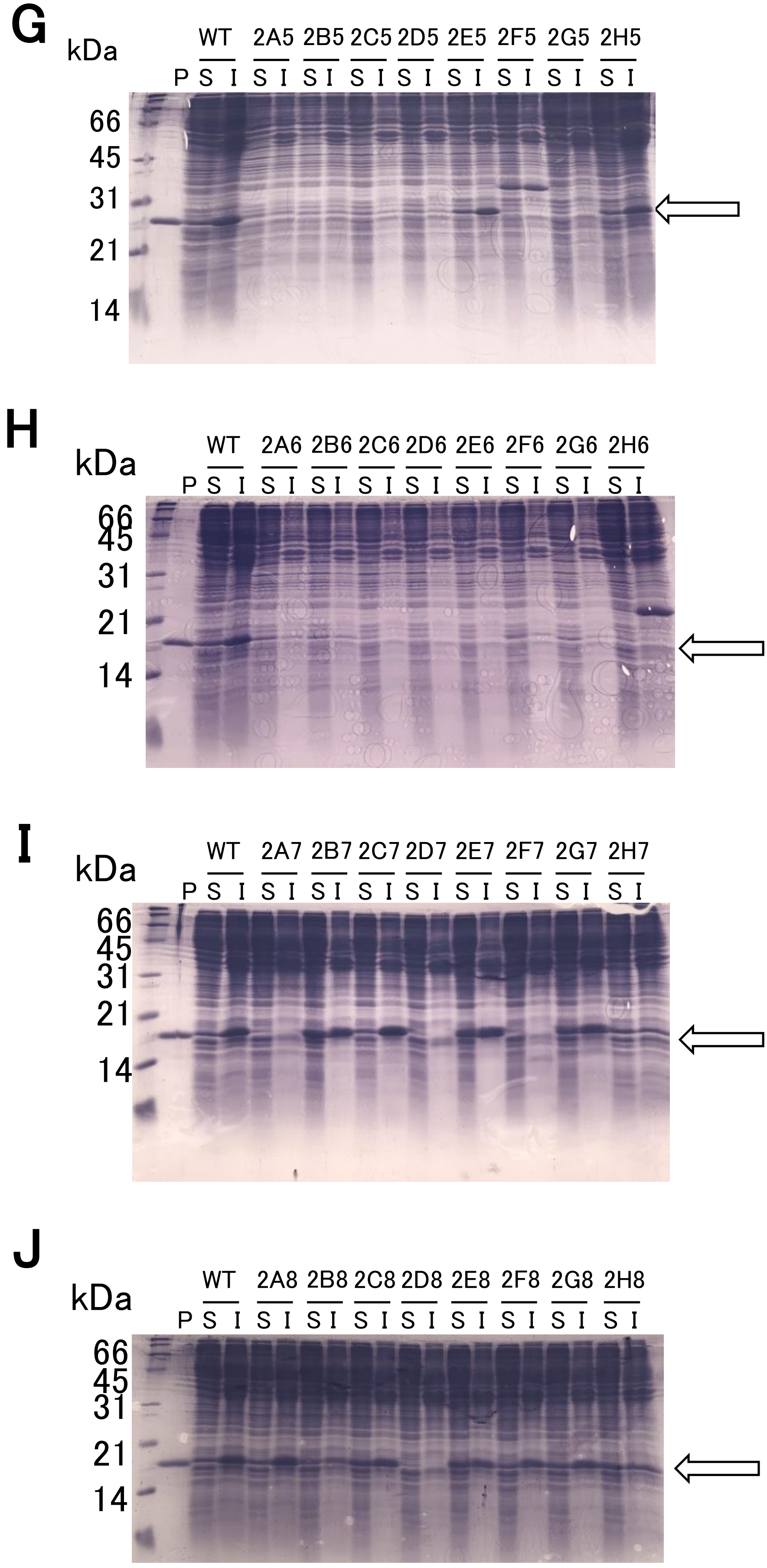
G
kDa
WT
2A5
2B5
2C5
2D5
2E5
2F5
2G5
2H5
P S I S I S I S I S I S I S I S I S I
66
45
31
21
14
H
kDa
WT
2A6
2B6
2C6
2D6
2E6
2F6
2G6
2H6
P S I S I S I S I S I S I S I S I S I
66
45
31
21
14
I
kDa
WT
2A7
2B7
2C7
2D7
2E7
2F7
2G7
2H7
P S I S I S I S I S I S I S I S I S I
66
45
31
21
14
J
kDa
WT
2A8
2B8
2C8
2D8
2E8
2F8
2G8
2H8
P S I S I S I S I S I S I S I S I S I
66
45
31
21
14

Fig. S3

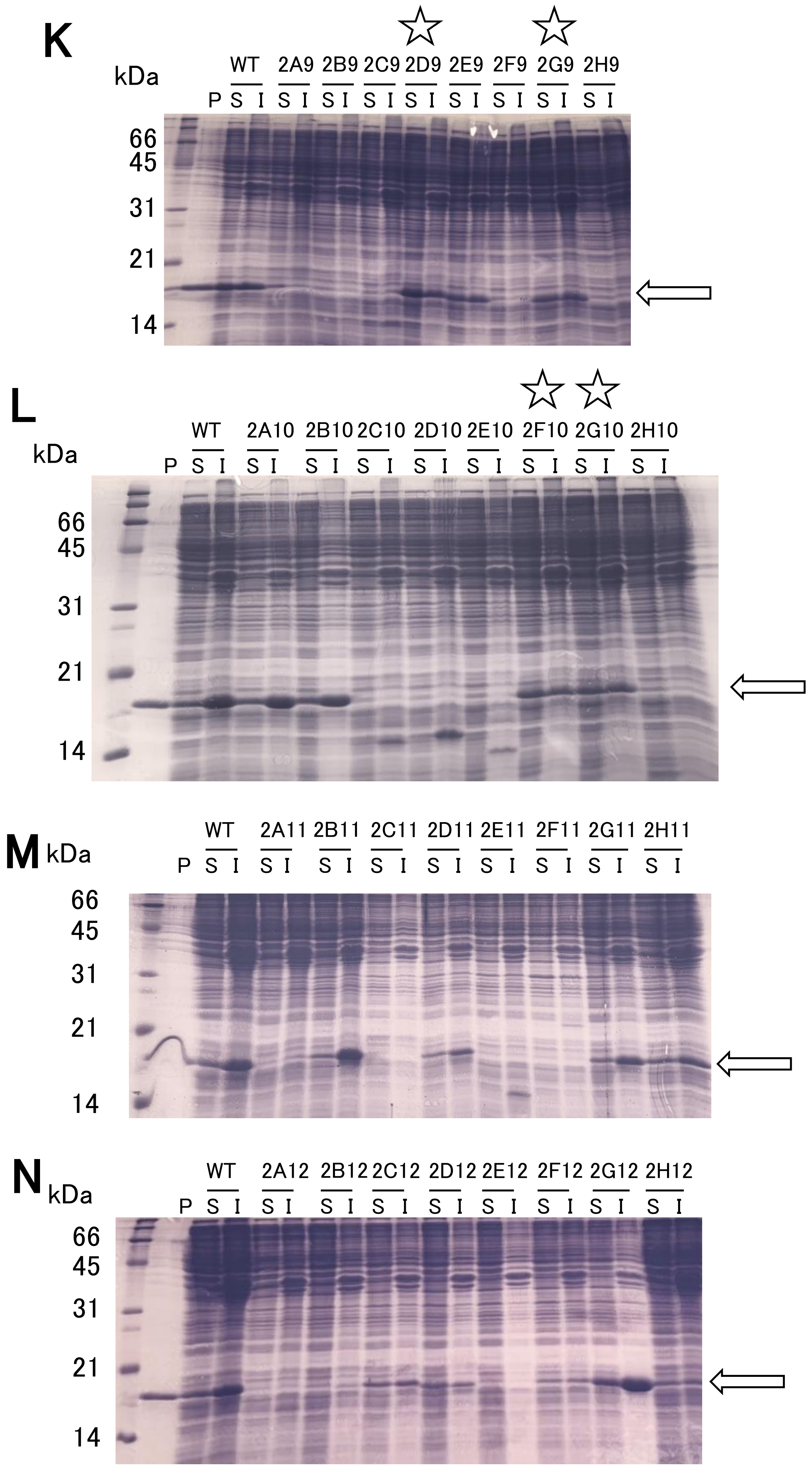
K
kDa
WT 2A9 2B9 2C9 2D9 2E9 2F9 2G9 2H9
P S I S I S I S I S I S I S I S I S I
66
45
31
21
14
L
kDa
WT 2A10 2B10 2C10 2D10 2E10 2F10 2G10 2H10
P S I S I S I S I S I S I S I S I S I
66
45
31
21
14
M
kDa
WT 2A11 2B11 2C11 2D11 2E11 2F11 2G11 2H11
P S I S I S I S I S I S I S I S I S I
66
45
31
21
14
N
kDa
WT 2A12 2B12 2C12 2D12 2E12 2F12 2G12 2H12
P S I S I S I S I S I S I S I S I S I
66
45
31
21
14

Fig. S3

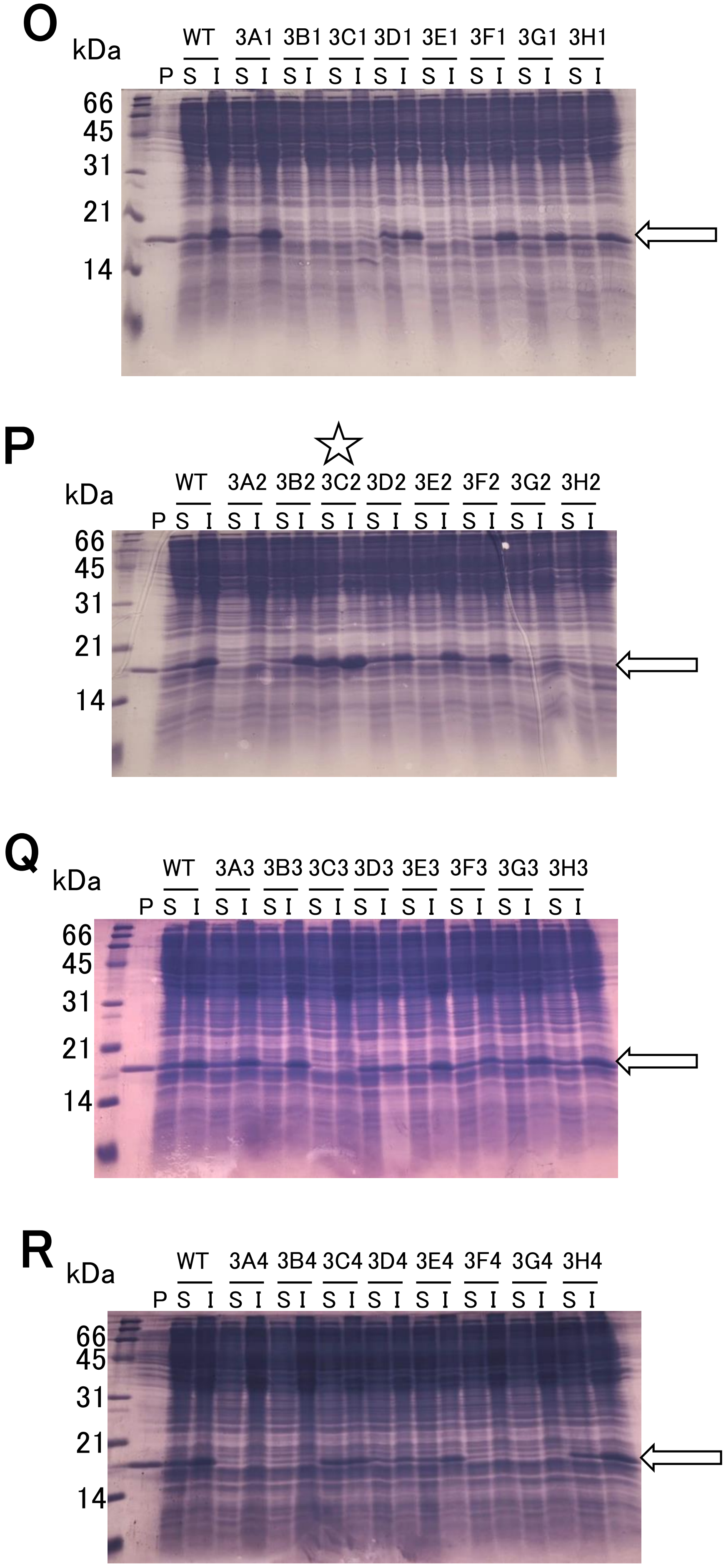
O
kDa
WT 3A1 3B1 3C1 3D1 3E1 3F1 3G1 3H1
P S I S I S I S I S I S I S I S I S I
66
45
31
21
14
P
☆
kDa
WT 3A2 3B2 3C2 3D2 3E2 3F2 3G2 3H2
P S I S I S I S I S I S I S I S I S I
66
45
31
21
14
Q
kDa
WT 3A3 3B3 3C3 3D3 3E3 3F3 3G3 3H3
P S I S I S I S I S I S I S I S I S I
66
45
31
21
14
R
kDa
WT 3A4 3B4 3C4 3D4 3E4 3F4 3G4 3H4
P S I S I S I S I S I S I S I S I S I
66
45
31
21
14

Fig. S3

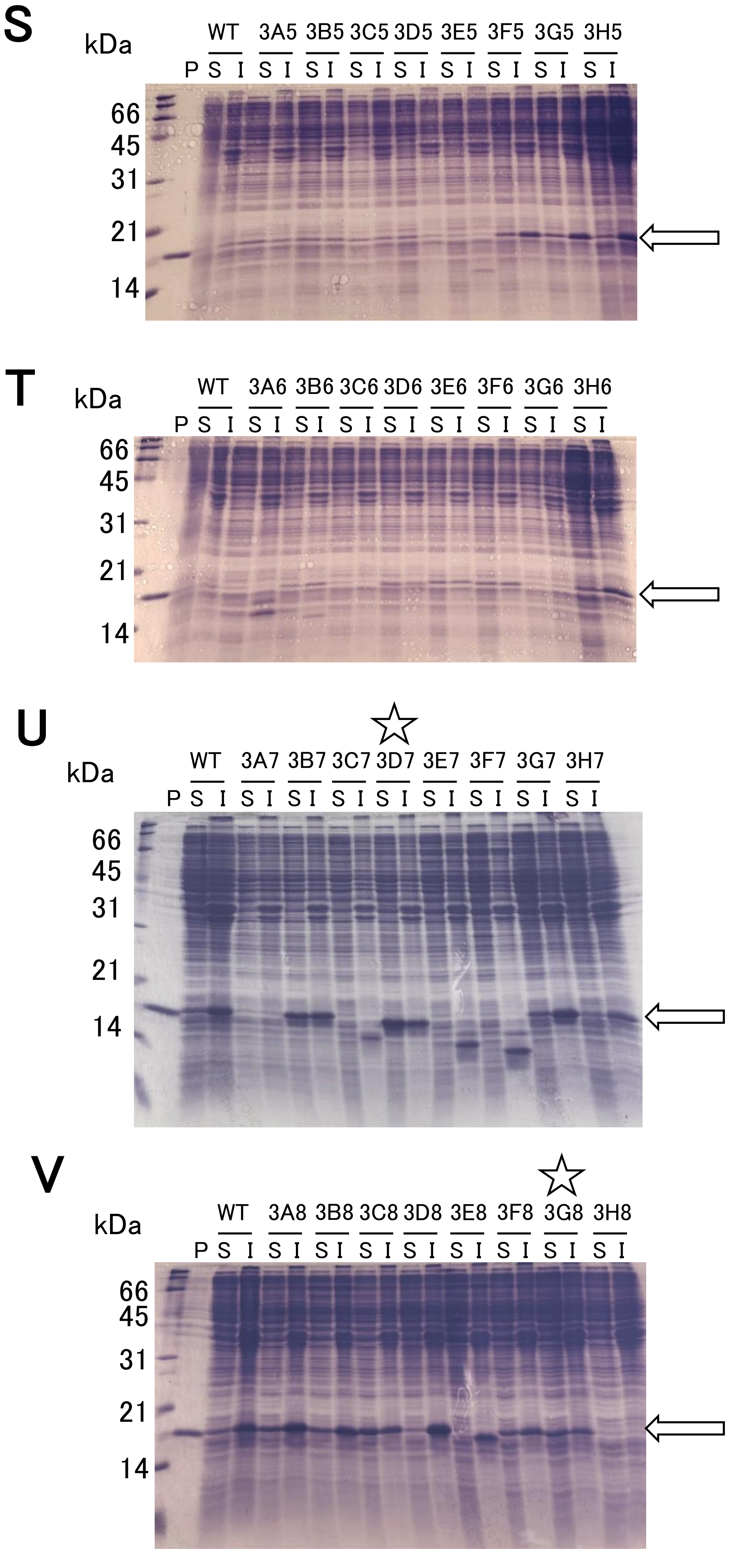
S
kDa
WT 3A5 3B5 3C5 3D5 3E5 3F5 3G5 3H5
P S I S I S I S I S I S I S I S I S I
66
45
31
21
14
T
kDa
WT 3A6 3B6 3C6 3D6 3E6 3F6 3G6 3H6
P S I S I S I S I S I S I S I S I S I
66
45
31
21
14
U
kDa
WT 3A7 3B7 3C7 3D7 3E7 3F7 3G7 3H7
P S I S I S I S I S I S I S I S I S I
66
45
31
21
14
V
kDa
WT 3A8 3B8 3C8 3D8 3E8 3F8 3G8 3H8
P S I S I S I S I S I S I S I S I S I
66
45
31
21
14

Fig. S3

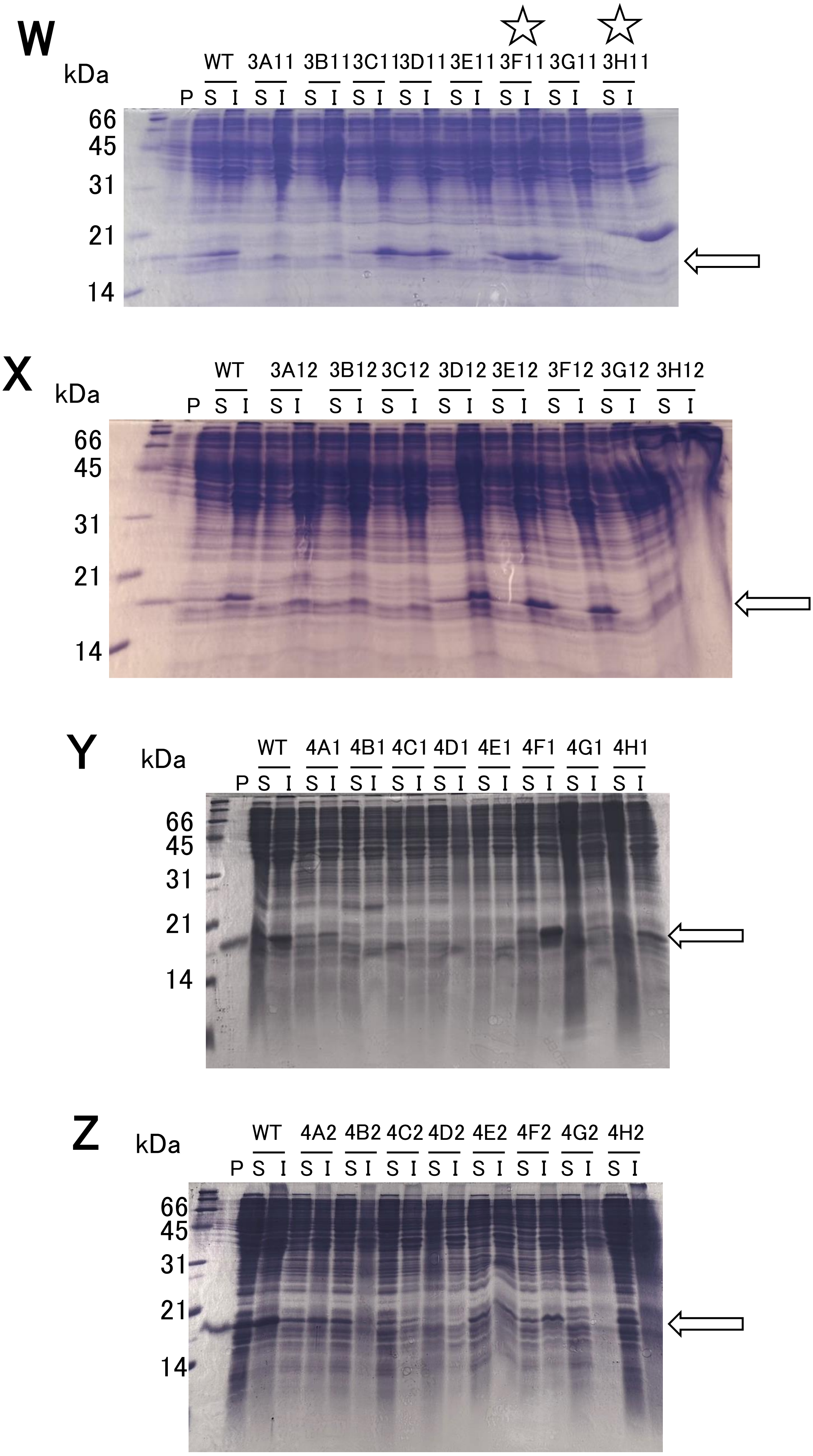
W
kDa
WT 3A11 3B11 3C11 3D11 3E11 3F11 3G11 3H11
P S I S I S I S I S I S I S I S I S I
66
45
31
21
14
X
kDa
WT 3A12 3B12 3C12 3D12 3E12 3F12 3G12 3H12
P S I S I S I S I S I S I S I S I S I
66
45
31
21
14
Y
kDa
WT 4A1 4B1 4C1 4D1 4E1 4F1 4G1 4H1
P S I S I S I S I S I S I S I S I S I
66
45
31
21
14
Z
kDa
WT 4A2 4B2 4C2 4D2 4E2 4F2 4G2 4H2
P S I S I S I S I S I S I S I S I S I
66
45
31
21
14

Fig. S3

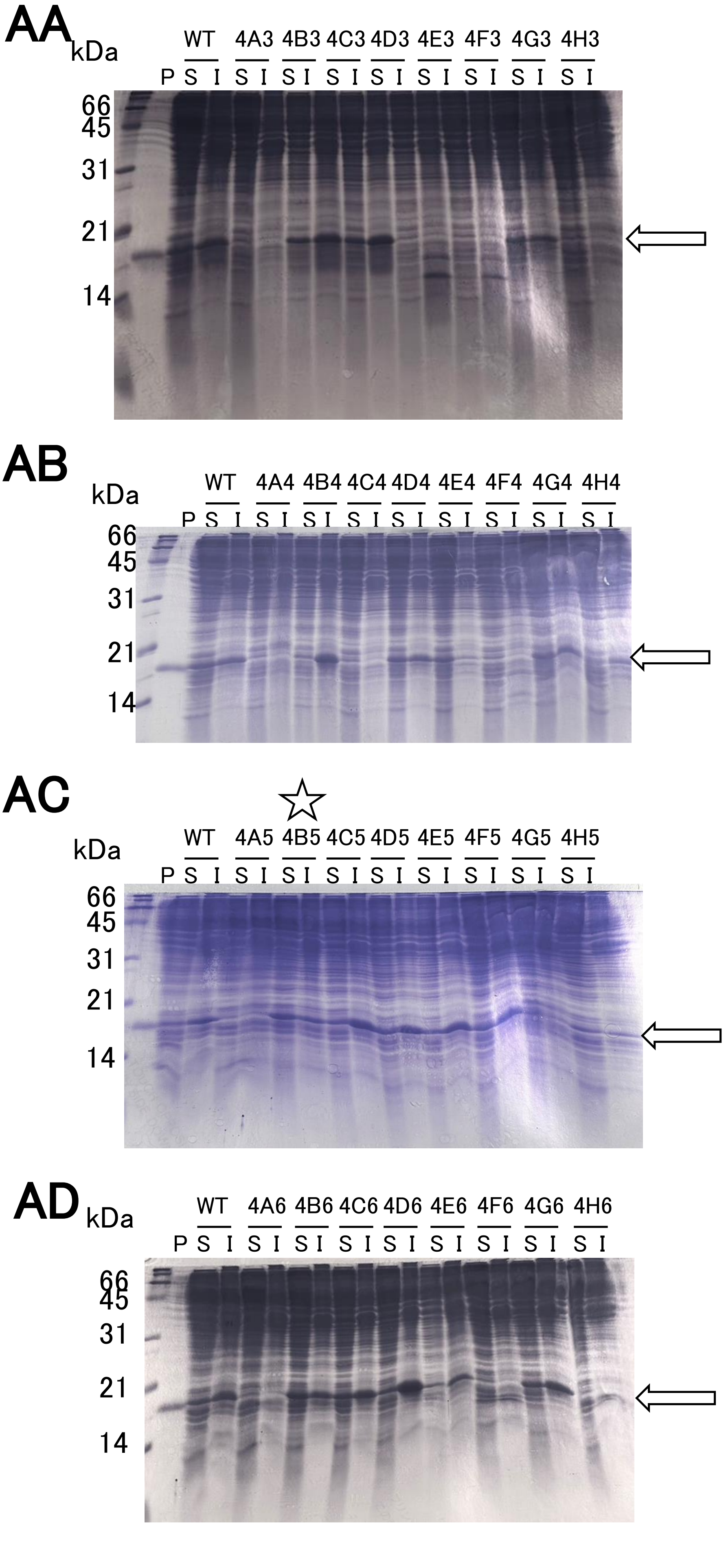
AA
kDa
WT 4A3 4B3 4C3 4D3 4E3 4F3 4G3 4H3
P S I S I S I S I S I S I S I S I S I
66
45
31
21
14
AB
kDa
WT 4A4 4B4 4C4 4D4 4E4 4F4 4G4 4H4
P S I S I S I S I S I S I S I S I S I
66
45
31
21
14
AC
☆
kDa
WT 4A5 4B5 4C5 4D5 4E5 4F5 4G5 4H5
P S I S I S I S I S I S I S I S I S I
66
45
31
21
14
AD
kDa
WT 4A6 4B6 4C6 4D6 4E6 4F6 4G6 4H6
P S I S I S I S I S I S I S I S I S I
66
45
31
21
14

Fig. S3

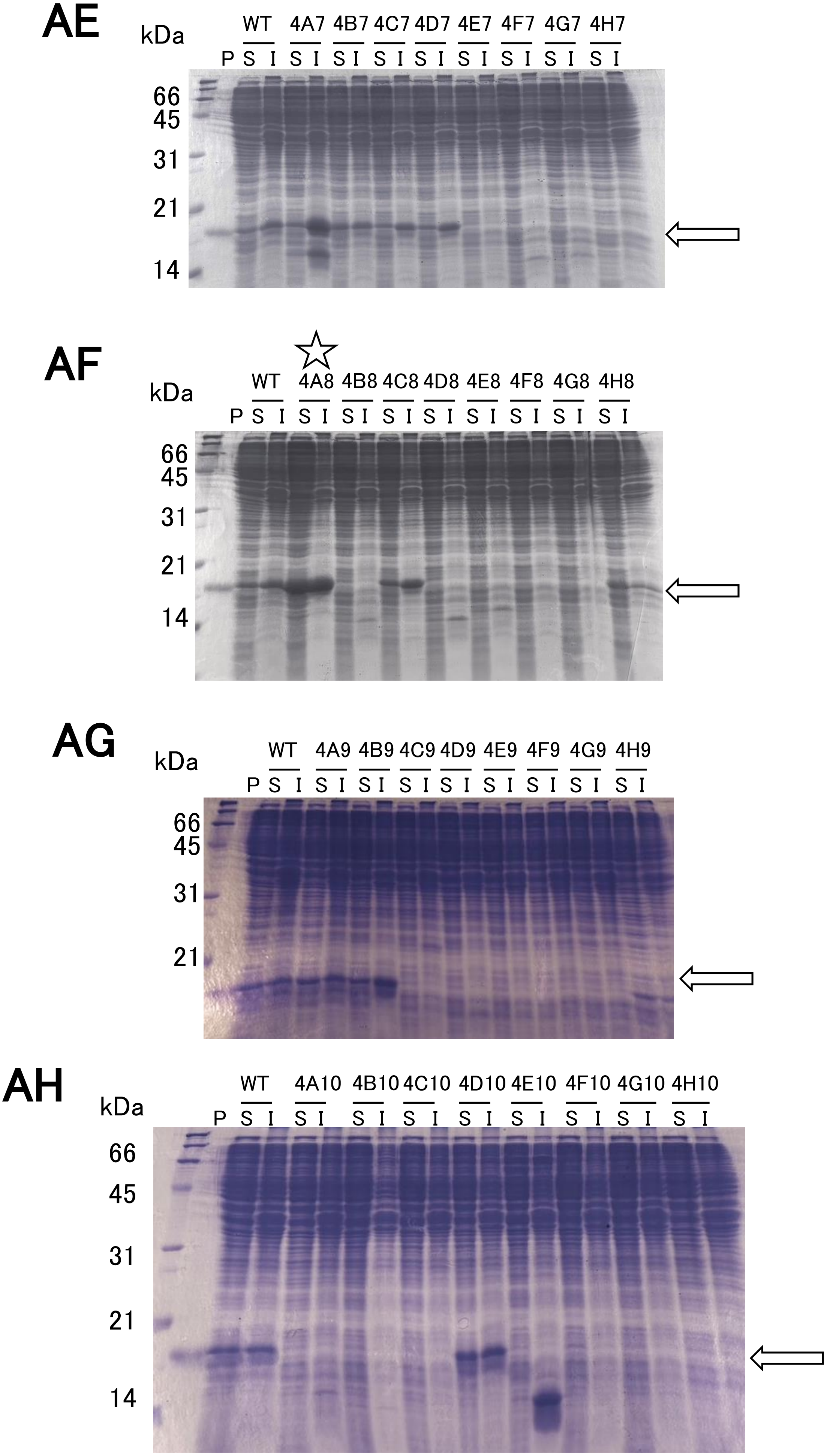
AE
kDa
WT 4A7 4B7 4C7 4D7 4E7 4F7 4G7 4H7
P S I S I S I S I S I S I S I S I S I
66
45
31
21
14
AF
kDa
WT 4A8 4B8 4C8 4D8 4E8 4F8 4G8 4H8
P S I S I S I S I S I S I S I S I S I
66
45
31
21
14
AG
kDa
WT 4A9 4B9 4C9 4D9 4E9 4F9 4G9 4H9
P S I S I S I S I S I S I S I S I S I
66
45
31
21
AH
kDa
WT 4A10 4B10 4C10 4D10 4E10 4F10 4G10 4H10
P S I S I S I S I S I S I S I S I S I
66
45
31
21
14

Fig. S3

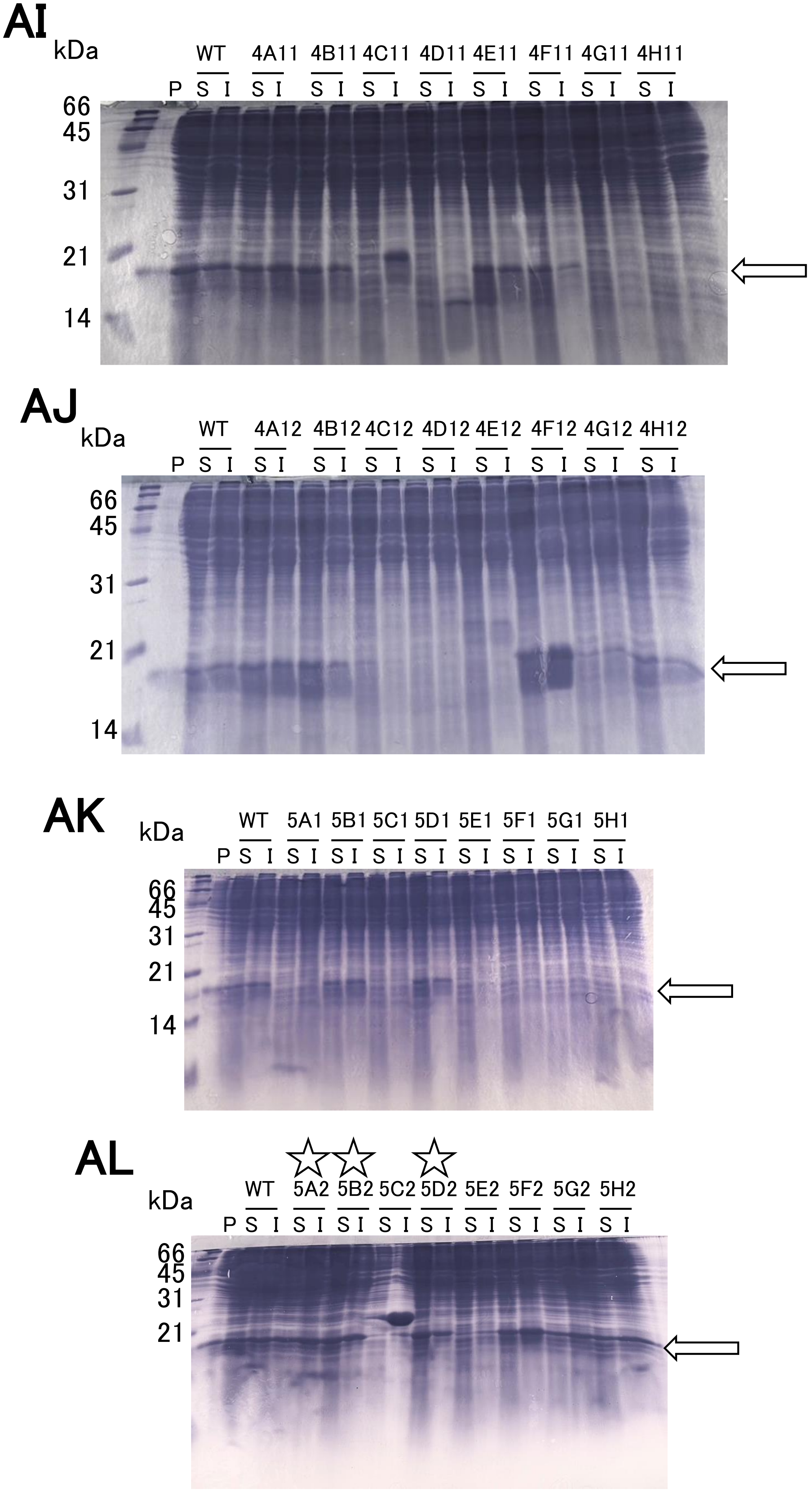
AI
kDa
WT 4A11 4B11 4C11 4D11 4E11 4F11 4G11 4H11
P S I S I S I S I S I S I S I S I S I
66
45
31
21
14
AJ
kDa
WT 4A12 4B12 4C12 4D12 4E12 4F12 4G12 4H12
P S I S I S I S I S I S I S I S I S I
66
45
31
21
14
AK
kDa
WT 5A1 5B1 5C1 5D1 5E1 5F1 5G1 5H1
P S I S I S I S I S I S I S I S I S I
66
45
31
21
14
AL
kDa
WT 5A2 5B2 5C2 5D2 5E2 5F2 5G2 5H2
P S I S I S I S I S I S I S I S I S I
66
45
31
21

Fig. S3

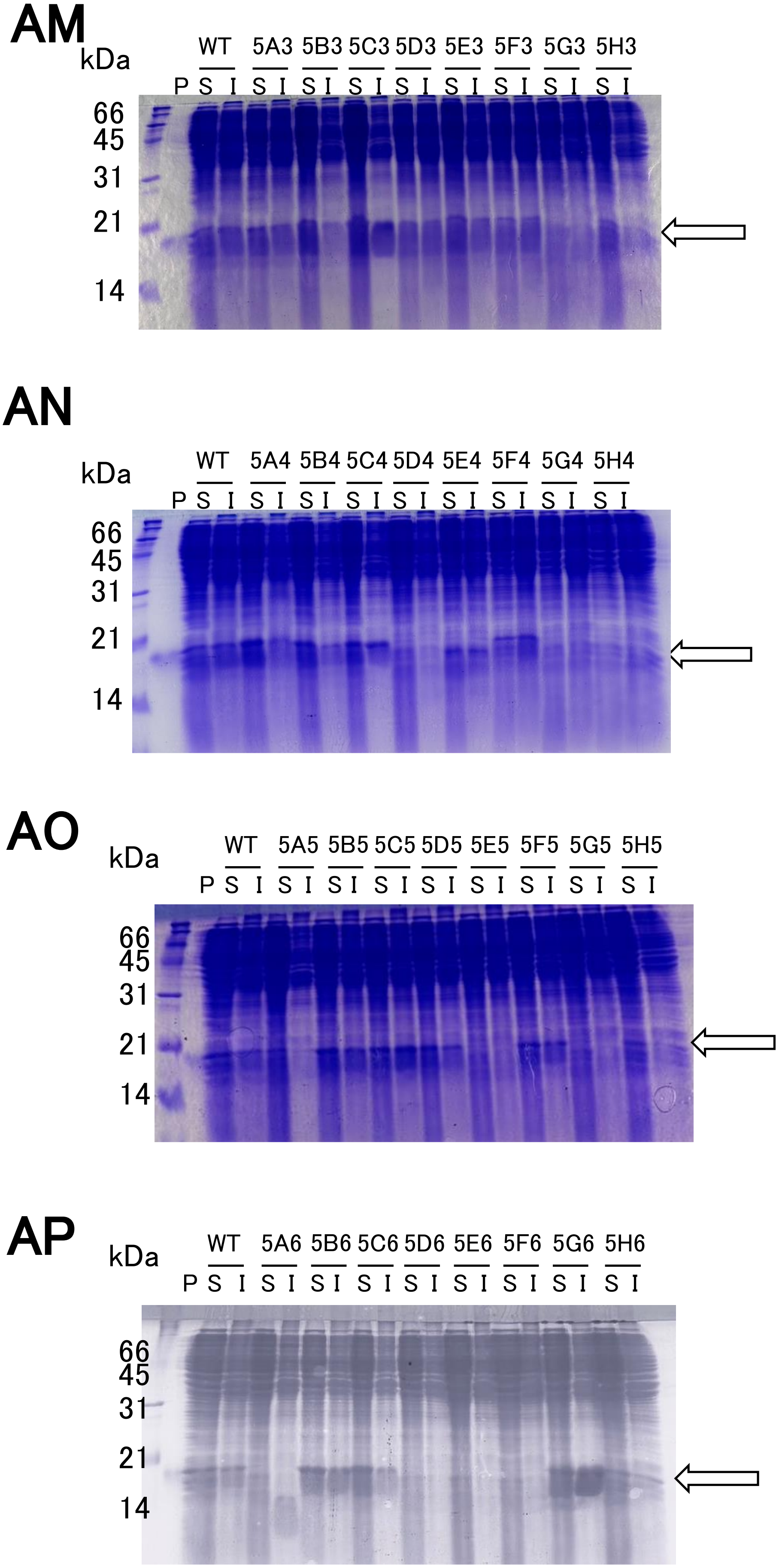
AM
kDa
WT 5A3 5B3 5C3 5D3 5E3 5F3 5G3 5H3
P S I S I S I S I S I S I S I S I S I
66
45
31
21
14
AN
kDa
WT 5A4 5B4 5C4 5D4 5E4 5F4 5G4 5H4
P S I S I S I S I S I S I S I S I S I
66
45
31
21
14
AO
kDa
WT 5A5 5B5 5C5 5D5 5E5 5F5 5G5 5H5
P S I S I S I S I S I S I S I S I S I
66
45
31
21
14
AP
kDa
WT 5A6 5B6 5C6 5D6 5E6 5F6 5G6 5H6
P S I S I S I S I S I S I S I S I S I
66
45
31
21
14

Fig. S3

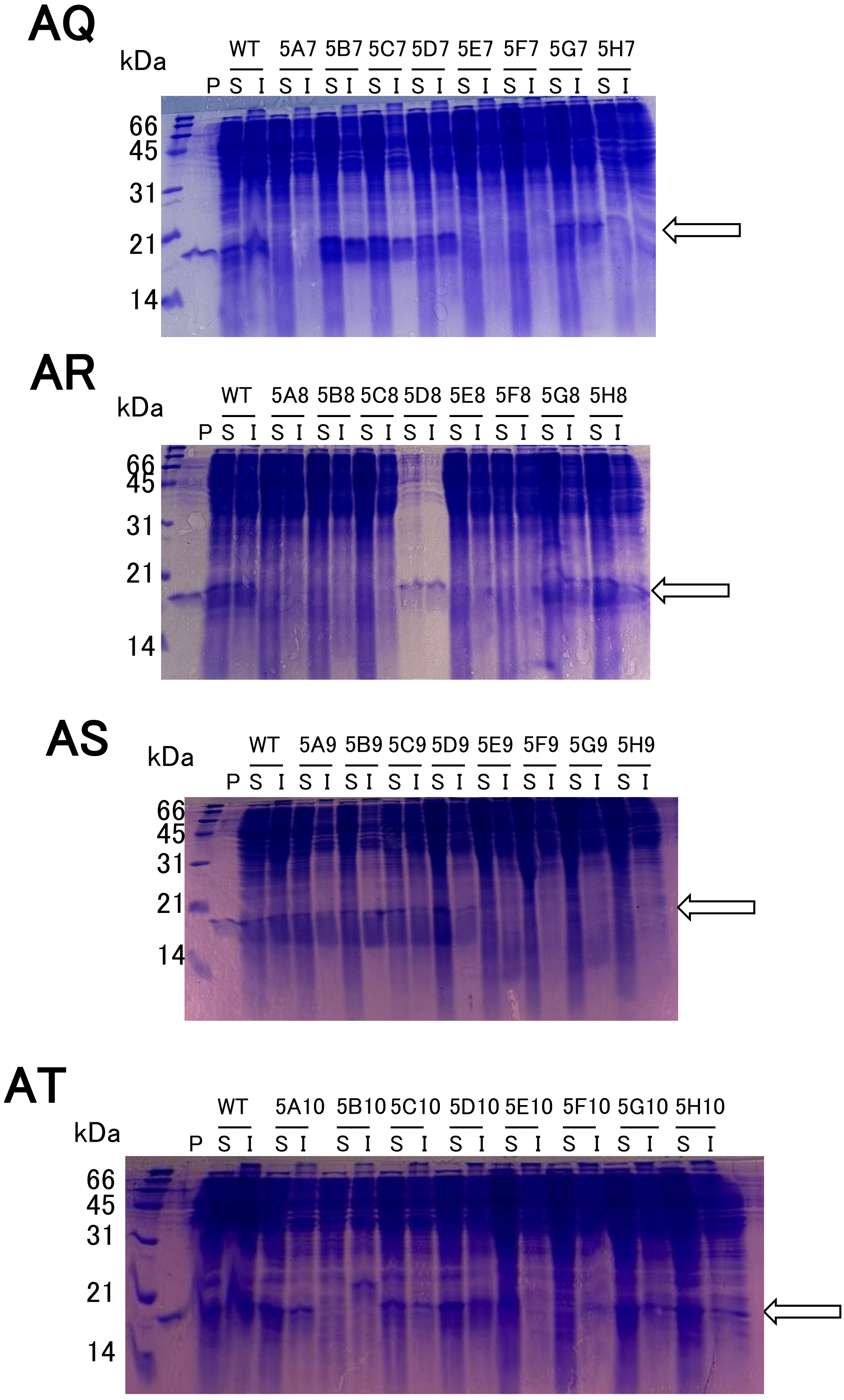
AQ
kDa
WT 5A7 5B7 5C7 5D7 5E7 5F7 5G7 5H7
P S I S I S I S I S I S I S I S I S I
66
45
31
21
14
AR
kDa
WT 5A8 5B8 5C8 5D8 5E8 5F8 5G8 5H8
P S I S I S I S I S I S I S I S I S I
66
45
31
21
14
AS
kDa
WT 5A9 5B9 5C9 5D9 5E9 5F9 5G9 5H9
P S I S I S I S I S I S I S I S I S I
66
45
31
21
14
AT
kDa
WT 5A10 5B10 5C10 5D10 5E10 5F10 5G10 5H10
P S I S I S I S I S I S I S I S I S I
66
45
31
21
14

Fig. S3

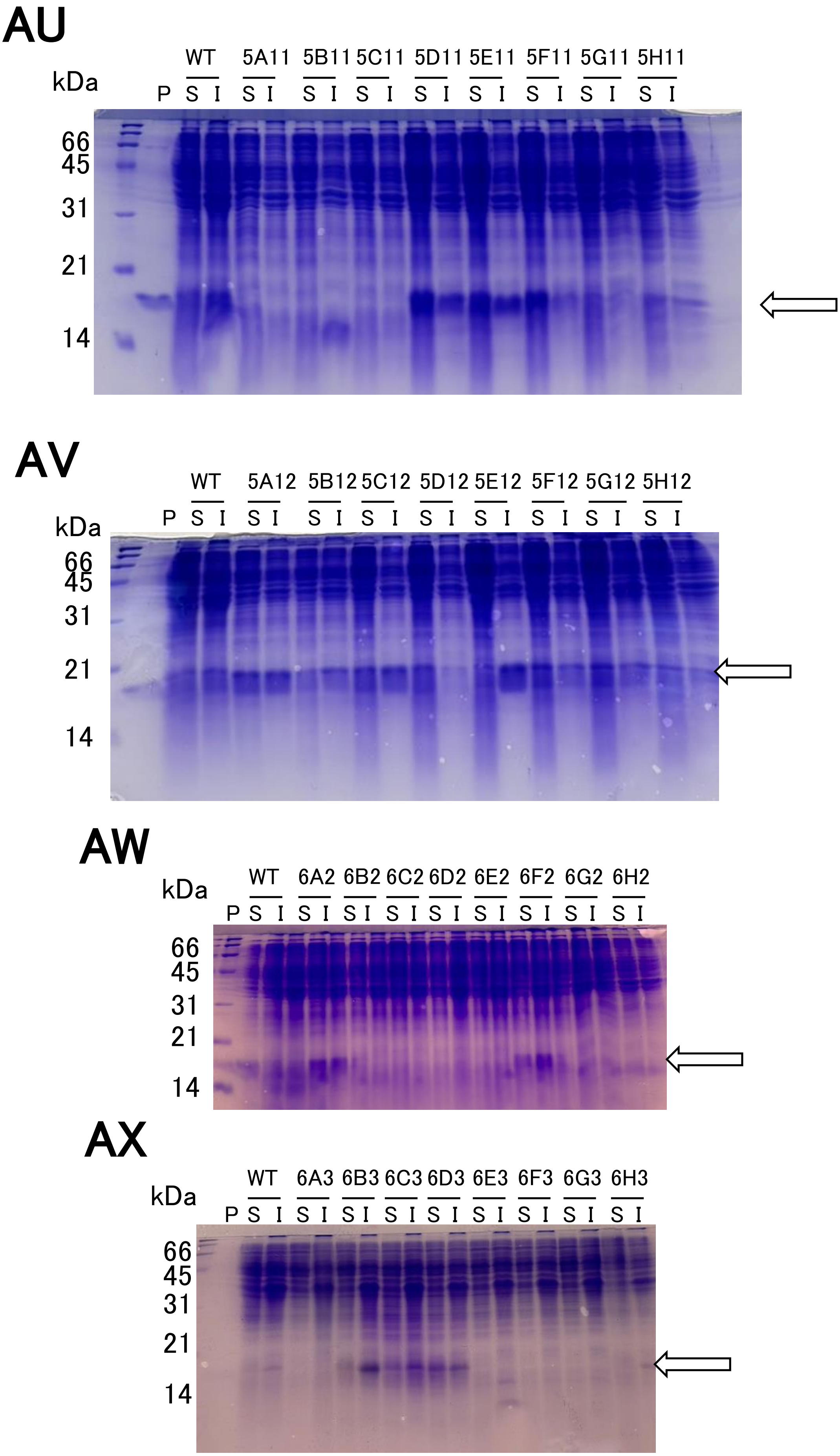
AU
WT
5A11
5B11
5C11
5D11
5E11
5F11
5G11
5H11
kDa
P S I S I S I S I S I S I S I S I S I
66
45
31
21
14
AV
WT
5A12
5B12
5C12
5D12
5E12
5F12
5G12
5H12
kDa
P S I S I S I S I S I S I S I S I S I
66
45
31
21
14
AW
WT
6A2
6B2
6C2
6D2
6E2
6F2
6G2
6H2
kDa
P S I S I S I S I S I S I S I S I S I
66
45
31
21
14
AX
WT
6A3
6B3
6C3
6D3
6E3
6F3
6G3
6H3
kDa
P S I S I S I S I S I S I S I S I S I
66
45
31
21
14

Fig. S3

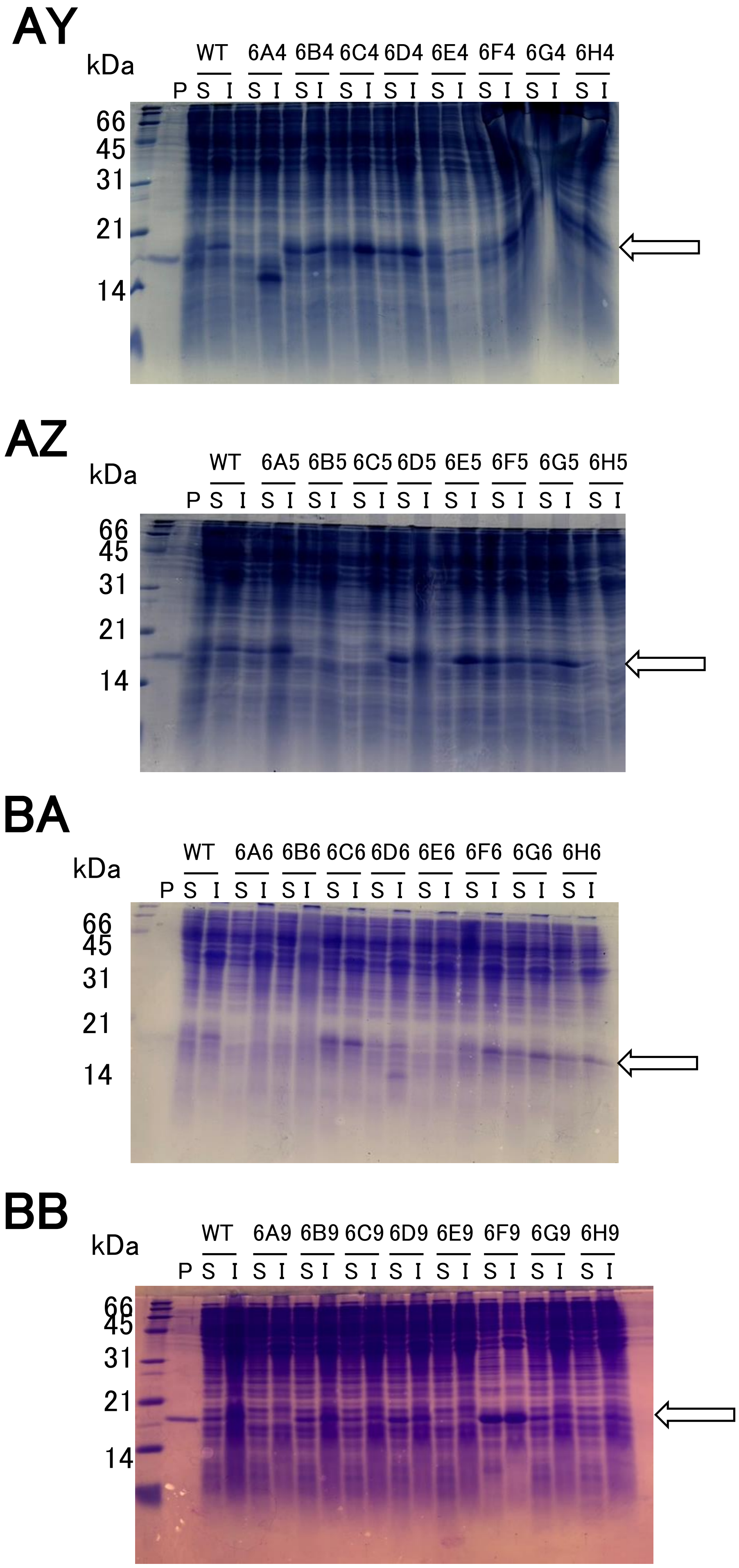
AY
kDa
WT 6A4 6B4 6C4 6D4 6E4 6F4 6G4 6H4
P S I S I S I S I S I S I S I S I S I
66
45
31
21
14
AZ
kDa
WT 6A5 6B5 6C5 6D5 6E5 6F5 6G5 6H5
P S I S I S I S I S I S I S I S I S I
66
45
31
21
14
BA
kDa
WT 6A6 6B6 6C6 6D6 6E6 6F6 6G6 6H6
P S I S I S I S I S I S I S I S I S I
66
45
31
21
14
BB
kDa
WT 6A9 6B9 6C9 6D9 6E9 6F9 6G9 6H9
P S I S I S I S I S I S I S I S I S I
66
45
31
21
14

Fig. S3

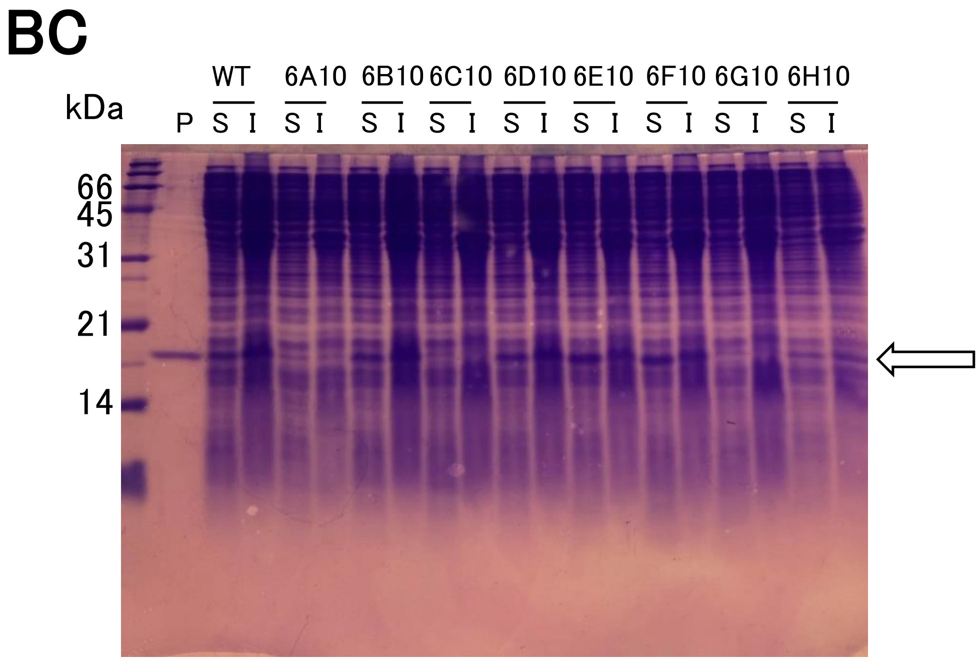

Fig. S3. Solubility of uvsY variants.
Coomassie Brilliant Blue-stained 15% polyacrylamide gels are shown. S and I indicate soluble and insoluble fractions of the extract of the BL21(DE3) cells transformed with uvsY variants expression plasmid and cultured at 20°C for 24 h after IPTG induction. The variants whose amounts in the soluble fraction were comparable to or more than that in the insoluble fraction are marked with a star.
